# University of Maryland

**Published:** 1846-05

**Authors:** 


### UNIVERSITY OF MARYLAND.

We have received the annual catalogue of students who attended lectures in the medical department of this institution during the last winter, from which we learn that the number was 147. From the circular appended, it appears that the fees for tickets have been reduced from twenty dollars to fifteen, and as the number of professors is but six the total of expences for the lectures is but ninety dollars. It is announced, in a note, that Dr. Power, having received the appointment of Lecturer on the Theory and Practice of Medicine, will discharge the duties of that chair until Professor Bartlett, who is now in Europe, returns. Are we to understand from this that Professor Bartlett is expected to return to the school in Baltimore? Is he to be professor at the same time in the University of Maryland and Transylvania University?

